# Comparison of Three Tracheal Intubation Procedures Using Personal Protective Equipment, Direct and Video Laryngoscopes: An Open, Randomized, Parallel Clinical Trial

**DOI:** 10.5812/aapm-148208

**Published:** 2024-09-17

**Authors:** Dita Aditianingsih, Pryambodho Pryambodho, Jonathan Antonius Wibowo, El Nissi Leonard, Chrisella Annabelle

**Affiliations:** 1 Department of Anesthesiology and Intensive Care, Cipto Mangunkusumo Hospital, Faculty of Medicine, Universitas Indonesia, Jakarta, Indonesia

**Keywords:** COVID-19, Intubation, Personal Protective Equipment (PPE)

## Abstract

**Background:**

During the COVID-19 pandemic, severe respiratory failure is a life-threatening condition, and life-saving tracheal intubation is a high-risk aerosol- and droplet-generating procedure. It is crucial to protect healthcare workers without compromising patient safety during intubation. The use of personal protective equipment (PPE) and different types of laryngoscopes are measures to reduce the risk of infectious transmission that might impact the intubation process.

**Objectives:**

This study aimed to evaluate the effects of different levels of PPE and types of laryngoscopes on the duration of the intubation process and its success rate.

**Methods:**

We conducted an open, randomized, parallel clinical trial on non-COVID-19 adult patients scheduled for elective and emergency surgeries under general anesthesia from November 2021 to May 2022. Patients were divided into three groups: Group 1 was intubated using a video-guided laryngoscope with operators wearing level three PPE; group 2 was intubated using a direct laryngoscope with operators wearing level three PPE; and group 3 was intubated using a direct laryngoscope with operators wearing level two PPE. Intubation was performed by 2nd- and 3rd-year anesthesia residents.

**Results:**

The duration of intubation varied significantly among the groups, with Group 1 taking the longest time (P = 0.046). Group 3 had a higher success rate for first-attempt intubation (P = 0.056).

**Conclusions:**

The use of PPE and video-guided laryngoscopy had varying effects on the intubation procedure, with the most notable impact being on the overall length of intubation. Further research with a larger sample size is needed to validate these findings.

## 1. Background

Coronavirus disease-2019 (COVID-19) is caused by severe acute respiratory syndrome Coronavirus 2 (SARS-CoV-2). The World Health Organization (WHO) declared COVID-19 a pandemic in April 2020 (1). Coronavirus disease-2019 can lead to life-threatening complications, such as acute respiratory distress syndrome (ARDS), with approximately 2.3% of patients requiring endotracheal intubation for mechanical ventilation (1, 2). Besides treating respiratory failure, endotracheal intubation is frequently used in surgical scenarios with general anesthesia to ensure proper airway patency. While elective surgeries have been scaled back or suspended due to the pandemic, emergency procedures for COVID-19 patients, including endotracheal intubation, have continued.

The intubation procedure carries the potential to generate droplets and aerosol particles, posing a significant risk of infectious transmission to healthcare workers (3). During the SARS outbreak of 2002 - 2003, a previous study highlighted a six-fold increase in the risk of viral transmission to healthcare workers during intubation procedures (4). Intubation procedures follow hospital recommendations for utilizing personal protective equipment (PPE) to mitigate the risk of transmission to medical staff. Adhering to these protocols and using appropriate PPE can substantially reduce the risk of coronavirus transmission (3).

Protecting medical personnel is as important as ensuring the safety of patients undergoing endotracheal intubation. Using a video-guided laryngoscope for intubating COVID-19 patients offers several advantages, such as assisting in glottis visualization and increasing the distance between the operator and the patient’s mouth. However, some operators may lack proficiency with video laryngoscopes, potentially adding obstacles to the intubation procedure. Longer intubation times and multiple attempts increase the risk of adverse hypoxic events (5).

## 2. Objectives

This study aimed to assess the effects of three different tracheal intubation procedures—using different levels of PPE and types of laryngoscopes—on the intubation process during the pandemic, focusing primarily on the total duration of intubation and the success rate of first-attempt intubation.

## 3. Methods

This was an open, randomized, parallel clinical trial conducted on adult patients in the operating room at a university teaching hospital in Jakarta from November 2021 to May 2022. Sampling was performed after obtaining approval from the ethics committee, with Clinical Trial Code NCT05108584.

We included non-COVID-19 patients aged 18 - 60 years, with an ASA score of 1 - 3 and a Body Mass Index (BMI) below 30 kg/m², who underwent elective or emergency surgery under general anesthesia with endotracheal intubation. Exclusion criteria included patients with predicted difficult airways, cervical spine disorders, critical illness, pregnancy, and hemodynamic instability identified during preoperative assessments.

This study was conducted after patients provided informed consent for general anesthesia using endotracheal intubation. Pre-anesthesia assessments were performed by the anesthesia team one day before the surgery, evaluating the patient’s physical status based on the American Society of Anesthesiologists (ASA) classification and airway condition. The results of these assessments, along with the anesthesia and surgery plans, were reported to the anesthesiologist in charge of the operating room. The researchers did not influence any decisions. The study details were explained to the patients, who were then planned for intubation according to their assigned group. Patients who agreed to participate signed the informed consent form and were included in the study.

According to Whitehead et al., preliminary studies require at least ten samples per group, which implies that this study needed at least 30 samples divided among three groups (6). To account for potential dropouts, 39 subjects were recruited and randomized into different groups using research randomizer software. The groups were as follows: Group 1 received intubation using a video-guided laryngoscope with operators wearing level three PPE; group 2 received intubation using a direct laryngoscope with operators wearing level three PPE; and group 3 received intubation using a direct laryngoscope with operators wearing level two PPE. No blinding was applied in this study.

Before performing anesthesia, the anesthesiologists prepared the vital sign monitor, anesthesia machine, airway equipment, induction drugs, anesthesia gases, emergency medications, and an acrylic aerosol intubation box. According to the operating room checklist, the anesthesiologist explained the anesthesia and airway management plan. The intubation was carried out by a second-year anesthesiology resident, assisted by a third-year resident and supervised by the anesthesiologist on duty. The researcher oversaw the recording of time and events during intubation. The intubation operator wore the appropriate PPE based on patient group randomization, as notified by the researcher, and all jumpsuits and surgical gowns were properly fitted.

**Figure 1.**
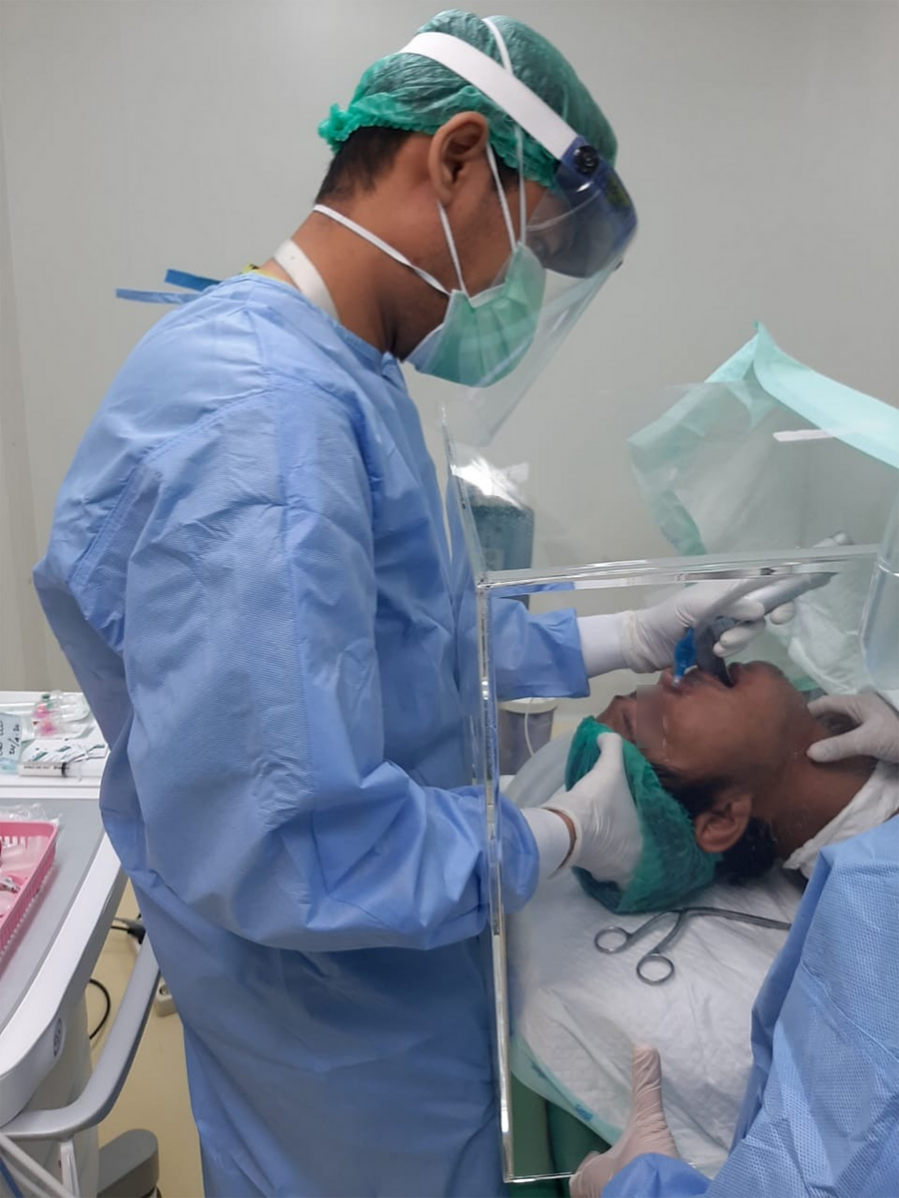
Intubation using personal protective equipment (PPE) during the Coronavirus disease-2019 (COVID-19) pandemic

Before intubation, the patient was placed on a standard monitor according to general anesthesia procedures. Once preparations were complete, the patient was positioned supine with their head propped up about 25 - 30° on a jelly donut-shaped pillow (Figure 1). Preoxygenation was conducted for three minutes using a face mask sized to fit the patient’s face, delivering six liters of oxygen per minute through a circuit breathing system, and this process was marked as point A. During this time, the assistant prepared the endotracheal tube with an introducer. After three minutes of preoxygenation, induction was performed using a modified rapid sequence intubation (RSI) technique. The drugs administered were Fentanyl (1.5 - 2 mcg/kg BW), Propofol (1 - 2 mg/kg BW), and Rocuronium (1.2 mg/kg BW) IV, marked as point B. Light ventilation was provided while waiting for the muscle-relaxing effect. Initial blood pressure was measured before intubation after drug administration. Laryngoscopy was performed 90 seconds after Rocuronium administration. The duration of laryngoscopy was measured from the removal of the face mask (point C) until the inflation of the endotracheal tube balloon and the end-tidal carbon dioxide (EtCO_2_) reading on the monitor (point D). Desaturation was defined as SpO_2_ below 95%, and positive pressure ventilation (PPV) was administered if needed until saturation exceeded 97%. The lowest saturation level and the timing of desaturation from point B were recorded. The depth of the endotracheal tube was confirmed by identifying pleural friction on bilateral lungs using ultrasound imaging, marked as point E. A second blood pressure measurement was taken after confirming the proper position of the endotracheal tube.

An assistive airway device could be used to aid the intubation process when necessary. During the intubation, any events were observed and recorded. Incidents that could occur included failure of the first laryngoscopy attempt, desaturation, difficulty requiring assistive airway devices, laryngoscopy requiring more than three attempts, change of airway operator, need for PPV with a bag-valve-mask, a 20% increase in post-intubation systolic blood pressure, and violations of PPE use. Additionally, operators evaluated glottis visualization levels using the Cormac-Lehane score, with a higher score indicating greater difficulty. The surgery proceeded after completing the intubation, provided that hemodynamics and ventilation were stable.

According to our hospital standards, level two PPE includes a surgical gown or apron, face shield, goggles or glasses, N95 mask, surgical mask, boots, scrubs, and two-layered gloves. Level three PPE, on the other hand, consists of a coverall jumpsuit (hazmat suit), face shield, goggles, N95 mask, surgical mask, boots, scrubs, and two-layered gloves.

Data were analyzed using SPSS version 21 software with the appropriate statistical tests. Primary characteristic data are displayed according to the type of variable. Numerical variables with a normal distribution are presented as means with standard deviations, and comparisons were made using the one-way ANOVA test. Numerical variables with a non-normal distribution are presented as medians with maximum and minimum values and compared using the Kruskal-Wallis test.

## 4. Results

**Figure 2.**
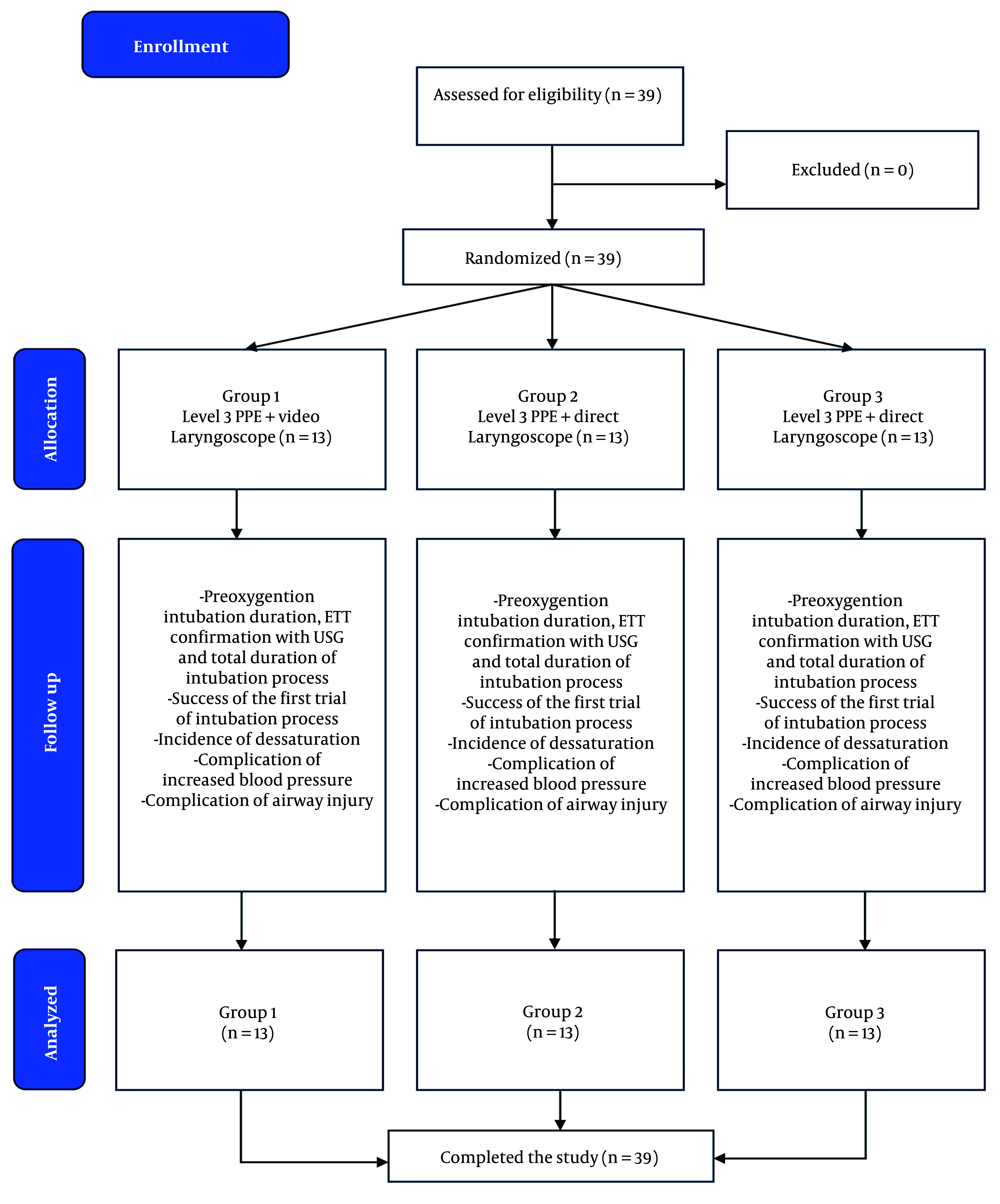
CONSORT flow diagram

Figure 2 illustrates the subject selection process. In this study, 39 subjects underwent an intubation procedure. The average age of the participants was 41.9 years, with the majority being female (66.6%). Most of the surgical procedures performed on the subjects were elective (97.4%), and the majority of subjects were classified as ASA 2 (82.1%).

Table 1 presents the comprehensive characteristics of the subjects in all three groups. The proportion of females was higher than that of males, and the number of patients classified as ASA 2 exceeded those classified as ASA 1 in each group. Only one emergency surgery, which was part of group 1, was included in the study. The other two groups consisted exclusively of patients undergoing elective surgeries.

**Table 1.** Baseline Characteristics ^a^

Characteristics	Level 3 PPE + Video Laryngoscope (Group 1)	Level 3 PPE + Direct Laryngoscopy (Group 2)	Level 2 PPE + Direct Laryngoscopy (Group 3)
**Age**	40.46 ± 12.0	41.07 ± 11.9	44.38 ± 8.94
**Gender**			
Male	6 (46.1)	5 (38.4)	2 (15.3)
Female	7 (53.8)	8 (61.5)	11 (84.6)
**Weight, kg**	59.9 ± 9.48	61.3 ± 9.82	56.6 ± 10.02
**Height, cm**	162.0 ± 9.61	162.7 ± 9.64	157.7 ± 6.39
**BMI, kg/m** ^**2**^	22.72 ± 2.96	23.04 ± 1.75	22.78 ± 3.73
**ASA**			
ASA 1	4 (30.7)	1 (7.6)	0 (0)
ASA 2	9 (69.2)	12 (92.3)	13 (100)
**Surgery type**			
Elective	12 (92.3)	13 (100)	13 (100)
Emergency	1 (7.6)	0 (0)	0 (0)

Abbreviation: BMI, Body Mass Index.

^a^ Values are expressed as mean ± SD or No. (%).

Table 2 compares the duration of each intubation process across all three groups. The data for preoxygenation duration (A), onset of neuromuscular inhibitor function (B-C), laryngoscopy duration (C-D), and total duration were non-normally distributed. In contrast, the duration for confirming endotracheal tube position with ultrasound (D-E) was normally distributed. The effect size analysis indicates minimal differences between the groups with statistically insignificant results. However, there is a significant difference in the total duration of the intubation process, with group 1 having the longest duration at 398 seconds (P = 0.046), followed by group 2 and then group 3.

**Table 2.** Comparison of Intubation Process Duration

Procedures	Level 3 PPE + Video Laryngoscope (Group 1)	Level 3 PPE + Direct Laryngoscope (Group 2)	Level 2 PPE + Direct Laryngoscope (Group 3)	P-Value	Effect Size
**Preoxygenation duration (A), sec ** ^**a**^	184 (180 - 237)	183 (180 - 230)	186 (182 - 209)	0.611	0.010
**Onset for neuromuscular inhibitor function (B-C), sec ** ^**a**^	93 (83 - 102)	94 (85 - 100)	92 (90 - 100)	0.584	0.013
**Laryngoscopy duration (C-D), sec ** ^**a**^	109 (34 - 360)	83 (31 - 440)	57 (40 - 204)	0.069	0.052
**Endotracheal tube position confirmation with USG (D-E), sec ** ^**b**^	14.69 ± 3.47	14.15 ± 3.02	14.69 ± 4.19	0.908	0.005
**Total Duration ** ^**a**^	398 (346 - 657)	368 (331 - 754)	352 (328 - 511)	0.046	0.058

^a^ Presented as median (range) with analysis using Kruskal Wallis.

^b^ Presented as mean ± standard deviation with analysis using one-way ANOVA.

Table 3 presents the distribution of different intubation outcomes across the groups. Although the differences were statistically insignificant, they reveal some interesting trends. Group 1 had a markedly lower success rate on the first attempt at laryngoscopy. In contrast, attempts exceeding three times were only necessary in group 2. Both groups 1 and 2 experienced desaturation incidents during intubation, with similar lowest SpO_2_ levels.

All three groups required larynx manipulation, but it was performed more frequently in group 3. The Cormac-Lehane scores indicated that groups 1 and 3 had similar favorable results, while group 2 had higher scores. Additionally, an increase in blood pressure after intubation was more commonly observed in groups 1 and 2.

**Table 3.** Intubation Process Outcome Comparison

Outcome	Level 3 PPE + Video Laryngoscope (Group 1)	Level 3 PPE + Direct Laryngoscope (Group 2)	Level 2 PPE + Direct Laryngoscope (Group 3)	P-Value
**Laryngoscopy success in one attempt ** ^**a**^	3 (23)	7 (53.8)	9 (69.2)	0.056
**Intubation attempts more than three times**	0 (0)	1 (7.6)	0 (0)	1.000
**Desaturation occurrence**	1 (7.6)	2 (15.3)	0 (0)	0.760
**Time of incident since B, sec ** ^**b**^	325	163.5 (145 - 182)	-	
**Lowest SpO**_**2**_ **, % ^**b**^**	90	89 (88-90)	-	
**Bougie use ** ^**c**^	0 (0)	1 (7.6)	0 (0)	1.000
**Operator change ** ^**c**^	1 (7.6)	1 (7.6)	0 (0)	1.000
**Need for PPV ** ^**c**^	1 (7.6)	1 (7.6)	0 (0)	1.000
**Larynx manipulation ** ^**a**^	3 (23)	6 (46.1)	8 (61.5)	0.174
**Cormac-Lehane score ** ^**c**^				0.297
1	9 (69.2)	5 (38.4)	9 (69.2)	
2	4 (30.7)	7 (53.8)	4 (30.7)	
3	0 (0)	1 (7.6)	0 (0)	
**Airway injury ** ^**c**^	2 (15.3)	5 (38.4)	4 (30.7)	0.550
**Systolic Blood Pressure Increase >20% post-intubation ** ^**c**^	2 (15.3)	1 (7.6)	0 (0)	0.760
**PPE violation ** ^**c**^	2 (15.3)	3 (23)	0 (0)	0.336

^a^ Presented in No. (%) along with Pearson’s chi-square analysis.

^b^ Data presented in median (range).

^c^ Presented in No. (%) along with Fisher exact test analysis.

**Figure 3.**
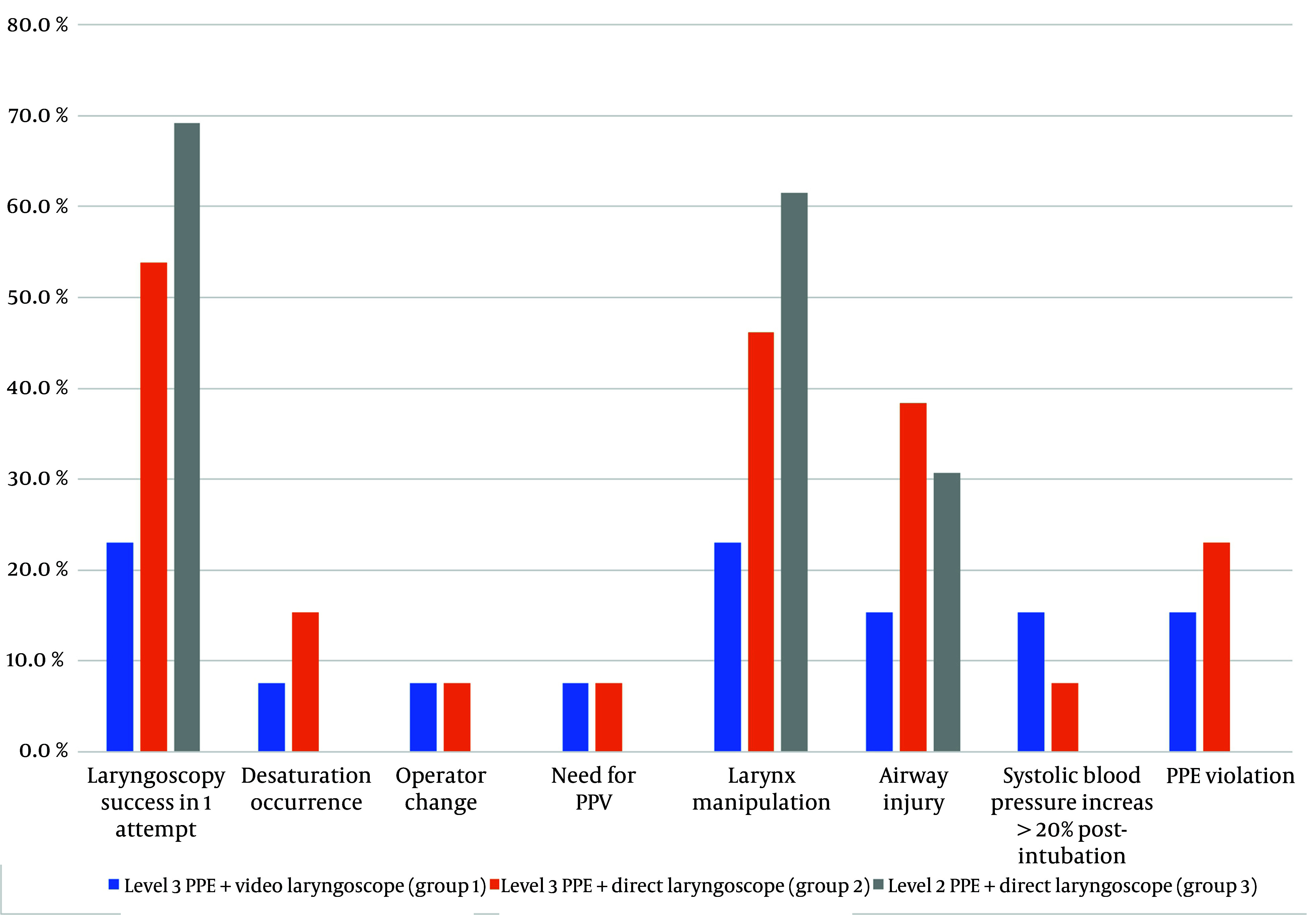
Intubation outcome comparison

Figure 3 shows that group 3, which used level two PPE and a direct laryngoscope, had a higher success rate for first-attempt laryngoscopy and intubation. This group also did not experience increased systolic blood pressure after intubation or any desaturation incidents. However, they required more frequent larynx manipulation compared to the other two groups.

## 5. Discussion

In this study, we found that the longest total duration of the intubation process was in group 1, while the shortest duration was in group 3. A systematic review by Sanfilippo et al. reported that intubation times were shorter with standard uniform compared to PPE (7). Previous research has highlighted the detrimental effects of PPE on vision and movement. Yánez Benítez et al. found that level three PPE could cause visual disturbances in 63% of cases, likely due to goggles, glasses, and coveralls reducing the field of view (8).

Additionally, bulky PPE can restrict normal movement, leading to slower maneuvers and awkward positions (9). Longer intubation durations increase the risk of desaturation in patients (10). Consistent with this, our study found desaturation events in groups 1 and 2 but not in group 3.

Laryngoscopy is an operator-dependent procedure, and familiarity with video-guided laryngoscopes can impact the success rate of first-attempt laryngoscopy. Direct laryngoscopes are routinely used in preoperative intubation procedures. Previous studies suggest that direct laryngoscopy often has better first-attempt success compared to video laryngoscopy, which requires specific visuospatial coordination skills (11). The additional challenges posed by higher-level PPE may further hinder performance. Despite this, our study showed that only group 2 required more than three intubation attempts, suggesting that the difficulty of level three PPE might be more constraining than the adaptability of operators to video laryngoscopes.

Visualization of the larynx and glottis is crucial for successful intubation. In our study, larynx manipulation to improve visualization was most frequently performed in group 3. Conversely, glottis visualization was most challenging in group 2, as indicated by the Cormac-Lehane score distribution. This suggests that the modality of laryngoscope has a greater impact on visualization than the level of PPE worn. Operators achieved similar glottis visualization with more uncomfortable higher-level PPE and a video-aided laryngoscope compared to the more comfortable level two PPE and a familiar laryngoscope. This indicates that the relationship between airway visualization and first-pass intubation success may not always be straightforward.

Incidents of airway injury occurred in all three groups. In this study, airway injury was identified by the presence of blood specks on the laryngoscope blades. It is theorized that using a video laryngoscope requires less force for intubation, potentially reducing the risk of trauma and injury to the airway (12). Conversely, the altered vision of operators wearing level three PPE and the greater force needed for the procedure resulted in higher rates of injury in group 2.

Post-intubation systolic blood pressure increases of more than 20% were observed only in groups wearing level three PPE. This is an important adverse event, as studies have linked post-intubation hypertension to major complications, including myocardial ischemia and re-rupture of aneurysmal subarachnoid hemorrhage. Another study found that repeated intubation attempts are significantly associated with a higher risk of post-intubation blood pressure elevation due to sympathetic stimulation during laryngoscopy (13). In our study, it can be speculated that groups 1 and 2, which had more than two to four intubation attempts, were affected, whereas group 3 had fewer attempts.

Personal protective equipment use violations were observed only in groups 1 and 2, which used level three PPE. These violations were likely related to issues with limited visualization, comfort, and maneuvering. Operators wearing goggles or face shields often experienced fogging, which made it difficult to perform the procedure safely. Additionally, the need to use assistive airway devices, such as bougies, also led to PPE violations, as operators had to remove the aerosol protector due to its shape, which obstructed access to the anterior neck. This issue is not unique to our study, as similar problems with PPE and airway procedures have been reported in other countries (14).

### 5.1. Study Limitations

This study is a single-center investigation with a small sample size, necessitating follow-up research to provide more definitive statistical insights. The study subjects were patients classified as ASA 1 or 2. In contrast, COVID-19 patients who require intubation typically present with respiratory failure, septic symptoms, and multi-organ system failure, making airway management more challenging. Therefore, the outcomes observed in this study may not fully represent the complexities of intubating COVID-19 patients.

### 5.2. Conclusions

The use of level three PPE and video-guided laryngoscopy impacted the intubation process during the COVID-19 pandemic. Significant differences in total intubation duration were observed among the three groups compared. Successful first-attempt intubation rates were higher in the group with operators wearing lower levels of PPE and using direct laryngoscopy. Additionally, desaturation occurred more frequently in groups with operators using higher levels of PPE, highlighting the need for more precautionary measures in these cases. Other important considerations include the likelihood of airway injury and increased blood pressure, which were higher in the groups using level three PPE.

## Data Availability

The dataset presented in the study is available on request from the corresponding author during submission or after publication.

## Clinical Trial Registration Code:

NCT05108584.
